# Effect of blastocyst morphology and developmental speed on transfer strategy for grade “C” blastocyst in vitrified‐warmed cycles

**DOI:** 10.1186/s13048-021-00798-w

**Published:** 2021-03-31

**Authors:** Yuxia He, Shiping Chen, Jianqiao Liu, Xiangjin Kang, Haiying Liu

**Affiliations:** 1 Department of Obstetrics and Gynecology, Center for Reproductive Medicine, Key Laboratory for Major Obstetric Diseases of Guangdong Province, The Third Affiliated Hospital of Guangzhou Medical University, Guangzhou, China; 2 Key Laboratory for Reproductive Medicine of Guangdong Province, The Third Affiliated Hospital of Guangzhou Medical University, 63 Duobao Road, Guangdong Guangzhou, China

**Keywords:** Poor‐quality blastocyst, Development speed, Morphology, Live birth rate, Neonatal outcomes

## Abstract

**Background:**

High-quality single blastocyst transfer (SBT) is increasingly recommended to patients because of its acceptable pregnancy outcomes and significantly reduced multiple pregnancy rate compared to double blastocyst transfer (DBT). However, there is no consensus on whether this transfer strategy is also suitable for poor-quality blastocysts. Moreover, the effect of the development speed of poor-quality blastocysts on pregnancy outcomes has been controversial. Therefore, this study aimed to explore the effects of blastocyst development speed and morphology on pregnancy and neonatal outcomes during the frozen embryo transfer (FET) cycle of poor-quality blastocysts and to ultimately provide references for clinical transfer strategies.

**Methods:**

A total of 2,038 FET cycles of poor-quality blastocysts from patients 40 years old or less were included from January 2014 to December 2019 and divided based on the blastocyst development speed and number of embryos transferred: the D5-SBT (*n* = 476), D5-DBT (*n* = 365), D6-SBT (*n* = 730), and D6-DBT (*n* = 467) groups. The SBT group was further divided based on embryo morphology: D5-AC/BC (*n* = 407), D5-CA/CB (*n* = 69), D6-AC/BC (*n* = 580), and D6-CA /CB (*n* = 150).

**Results:**

When blastocysts reach the same development speed, the live birth and multiple pregnancy rates of DBT were significantly higher than those of SBT. Moreover, there was no statistical difference in the rates of early miscarriage and live birth between the AC/BC and CA/CB groups. When patients in the SBT group were stratified by blastocyst development speed, the rates of clinical pregnancy (42.44 % vs. 20.82 %) and live birth (32.35 % vs. 14.25 %) of D5-SBT group were significantly higher than those of D6-SBT group. Furthermore, for blastocysts in the same morphology group (AC/BC or CA/CA group), the rates of clinical pregnancy and live birth in the D5 group were also significantly higher than those of D6 group.

**Conclusions:**

For poor-quality D5 blastocysts, SBT can be recommended to patients because of acceptable pregnancy outcomes and significantly reduced multiple pregnancy rate compared with DBT. For poor-quality D6, the DBT strategy is recommended to patients to improve pregnancy outcomes. When blastocysts reach the same development speed, the transfer strategy of selecting blastocyst with inner cell mass “C” or blastocyst with trophectoderm “C” does not affect the pregnancy and neonatal outcomes.

## Introduction

The *in vitro* fertilization–embryo transfer (IVF-ET) technology is widely used worldwide and benefits many couples with infertility. However, multiple embryo transfer resulting from this technology led to an increased incidence of multiple pregnancy, which is considered the most common adverse event associated with the IVF-ET technology [1]. Multiple pregnancy is associated with an increased risk of maternal and fetal complications, including miscarriage, preeclampsia, hypertension, preterm labor, and perinatal morbidity and mortality [2, 3]. Therefore, employing the strategy of single embryo transfer is an effective measure to minimize the incidence of multiple pregnancy for infertile patients undergoing assisted reproductive technology (ART).

Blastocyst transfer could yield higher implantation and clinical pregnancy rates compared with cleavage stage embryo transfer [4]. Moreover, single high-quality blastocyst transfer not only yields an acceptable pregnancy outcome compared to double blastocyst transfer (DBT) but also significantly reduces the incidence of multiple pregnancy [5–7]. Therefore, the practice of single high-quality blastocyst transfer is gradually accepted by physicians around the world, which could reduce maternal complications and improve perinatal outcomes. However, for patients achieving only poor-quality blastocysts, DBT is usually recommended to increase the live birth rate. The multiple pregnancy rate of double poor-quality blastocysts can still reach 33 % in frozen embryo transfer (FET) cycles [8]. Our previous studies showed that the multiple pregnancy rate of double poor-quality D5 blastocysts is as high as 30–50 %, and suggested that single poor-quality D5 blastocyst transfer can be recommended for patients only with grade “C” blastocyst, regardless of age [9]. However, this research did not mention whether the developmental potential of blastocysts with inner cell mass (ICM) “C” is different from that of blastocysts with trophectoderm (TE) “C” and the effect of different selection strategy on pregnancy and neonatal outcomes.

When blastocysts reach the same grade, it is unclear whether we should focus on the embryo development speed before transfer. A recent study illustrated the impact of embryo development speed on pregnancy outcomes based on blastocyst morphology [10]. Therefore, the strategy of blastocyst transfer should be determined according to the embryo development speed and morphology. To the best of our knowledge, there is no study assessed the effects of the number of blastocysts transferred, blastocyst morphology, and development speed on neonatal outcomes, solely in grade “C” blastocyst. Therefore, in the present study, we explored the influence of blastocyst morphology and development speed on vitrified-warmed blastocyst transfer pregnancy and neonatal outcomes and ultimately provided evidence for the implementation of poor-quality blastocyst transfer strategies.

## Materials and methods

### Study population and grouping

A retrospective study that included 2,038 FET cycles with poor-quality blastocysts was conducted at the Reproductive Medicine Center of the Third Affiliated Hospital of Guangzhou Medical University from January 2014 to December 2019. The inclusion criteria were as follows: (1) poor-quality blastocyst transfer; (2) age ≤ 40 years; and (3) endometrial thickness ≥ 7 mm. The exclusion criteria were as follows: (1) donated oocytes or embryos; (2) cycles with preimplantation genetic testing (PGT); (3) transferred blastocysts frozen on day 7; (4) stage III to IV endometriosis or adenomyosis; (5) known uterine anomalies including intrauterine adhesion, septal uterine cavity, endometrial polyps, and submucosal fibroid; (6) untreated hydrosalpinx; (7) uncontrolled endocrine and/or immune disorders or other systemic diseases, including hypertension, diabetes, thyroid disease, hyperprolactinemia, antiphospholipid syndrome, and systemic lupus erythematosus.

FET cycles were categorized into four groups according to blastocyst development speed and the number of embryos transferred, namely the D5-SBT group (*n* = 476), D5-DBT group (*n* = 365), D6-SBT group (*n* = 730), and D6-DBT group (*n* = 467). Patients in the SBT group were again divided into four subgroups based on the blastocyst morphology: D5-AC/BC (*n* = 407), D5-CA/CB (*n* = 69), D6-AC/BC (*n* = 580), and D6-CA /CB (*n* = 150). A total of 114 patients contributed multiple cycles during the study period. The study was approved by the local Ethics Committee of the Third Affiliated Hospital of Guangzhou Medical University.

### Laboratory protocol

Conventional IVF or ICSI was performed depending on semen parameters and previous fertilization histories. For IVF cycles, cumulus-oocyte complexes were inseminated with progressively motile spermatozoa in fertilization culture medium (G-IVFTM PLUS, Vitrolife, Sweden). Oocytes for ICSI were denuded 2–3 h after ovum pickup, and sperm microinjection was performed 5–6 h after oocytes retrieval. Fertilization was checked about 16 h post insemination/injection and was determined by the presence of two pronuclei (2PN). Embryos were placed into the incubator (K-MINC-1000, Cook, United States) and cultured at 6 % CO_2_, 5 % O_2_ and 37℃. G-1™ plus (Vitrolife, Sweden) was used for cleavage stage embryos and G-2™ plus (Vitrolife, Sweden) for blastocyst stage. The quality of day 3 cleavage embryo was assessed about 68 h post insemination/injection, and the quality of blastocysts were evaluated on Day 5 about 116 h post insemination/injection or Day 6 about 140 h post insemination/injection.

Blastocysts were scored according to the Gardner grading system, including degree of expansion and quality of the ICM and TE before freezing on day 5 or 6 by three embryologist with over 15 years of experience [11]. Briefly, blastocyst stage was graded 1 is an embryo in which the blastocoele occupies less than half of the volume; stage 2 is an embryo with the blastocoele occupied half of or greater than half of the volume of the embryo; stage 3 is a full blastocyst with the blastocoele completely filling the embryo; stage 4 is an expanded blastocyst with the blastocoele volume larger than that of the early embryo, with a thinning zona; stage 5 is a hatching blastocyst with the trophectoderm starting to herniate though the zona; and stage 6 is a hatched blastocyst in which the blastocyst has completely escaped from the zona. The inner cell mass was assessed as follows: A, tightly packed, many cells; B, loosely grouped, several cells; or C, very few cells. The trophectoderm was assessed as follows: A, many cells forming a cohesive epithelium; B, few cells forming a loose epithelium; or C, very few large cells. Blastocyst was recorded as high-quality embryo if they reached at least an expansion stage 3 with A or B for ICM and TE. The embryos included in this study were all poor-quality blastocysts, which are defined as at least an expansion stage 3 with ICM “C” or TE “C.” In all FET cycles, no more than two blastocysts were transferred.

### Vitrification and thawing

The precise vitrification and thawing protocol of the blastocysts was carried out according to the manufacturer’s instructions (Kitazato Biophama Co. Ltd. Shizuoka. Japan). For vitrification, an artificial shrinkage of the blastocoele was performed with laser. The blastocysts were equilibrated in an Equilibration Solution for 2 min and then placed into Vitrification Solution and remained for 45–60 s at 37℃. Then the blastocysts were transferred on the Cryotop strip and plunged into liquid nitrogen immediately. For thawing blastocyst, the top of the Cryotop containing the embryo was directly immersed into Thawing Solution for 1 min at 37℃. Next, blastocyst was transferred sequentially to a Diluent Solution, a Washing Solution 1, and then Washing Solution 2 where they respectively remained for 3-5-5 min per sequential step at room temperature. Then, the blastocysts were placed in blastocyst culture medium (K-SIBM, Cook) and cultured in an incubator at 37℃with 6 % CO_2_, 5 % O_2_ and 89 % N_2,_ then transferred about 4–5 h after thawing.

### Endometrial preparation for the FET cycle embryo transfer

Endometrial preparation for the FET cycle in this study was achieved using natural cycle (NC) or hormone replacement treatment (HRT) programs. In short, NC was applied for patients with regular menstrual cycles and ovulation. The ovulation in the NC protocol was determined by monitoring the follicular development with transvaginal ultrasonography and hormone levels. HRT was applicable for patients with irregular menstrual cycles or poor endometrium development in NC. The patients were treated with daily oral estradiol valerate tablets (Progynova, Bayer, Germany) from the second to the fourth day of menstruation. When endometrial thickness reached 7 mm or thicker, exogenous progesterone was administered daily.

One or two thawed blastocysts were transferred on the sixth day after ovulation or progesterone exposure using a soft-tipped Wallace (Portex Led., Hythe, United Kingdom) catheter under ultrasound guidance. All patients received luteal support with progesterone after embryo transfer and continued to 10 weeks of gestational age if a pregnancy occurred.

### Outcome parameters

The primary outcome of this study was the live birth rate (LBR). Secondary endpoints included rates of implantation, clinical pregnancy, multiple pregnancy, spontaneous miscarriage, and neonatal outcomes. Neonatal outcomes included preterm birth, birth weight, height, and low birth weight.

Live birth was defined as the delivery of any viable infant who was 28 weeks of gestation or older, and twins delivered by one mother were calculated as one live birth. Clinical pregnancy was defined as the presence of gestational sac transvaginal ultrasound at 6–8 weeks of gestation. Early miscarriage was defined as a spontaneous pregnancy demise at less than 12 weeks of gestation. Preterm birth was defined as a delivery before completing 37 weeks of gestation, and low birth weight was defined as a birth weight less than 2,500 g.

### Statistical analysis

The statistical analysis was performed with the use of the Statistical Package for Social Science (SPSS) version 22.0. The baseline characteristics were expressed as the mean ± standard deviation (SD), and differences in variables were compared using Student’s t-test or one-way analysis of variance (ANOVA). Categorical variables were described as frequencies and percentages and compared using the chi-square test and Fisher’s exact test when the number of events was less than 5. A *P-*value < 0.05 was considered statistically significant.

## Results

**Fig. 1:**
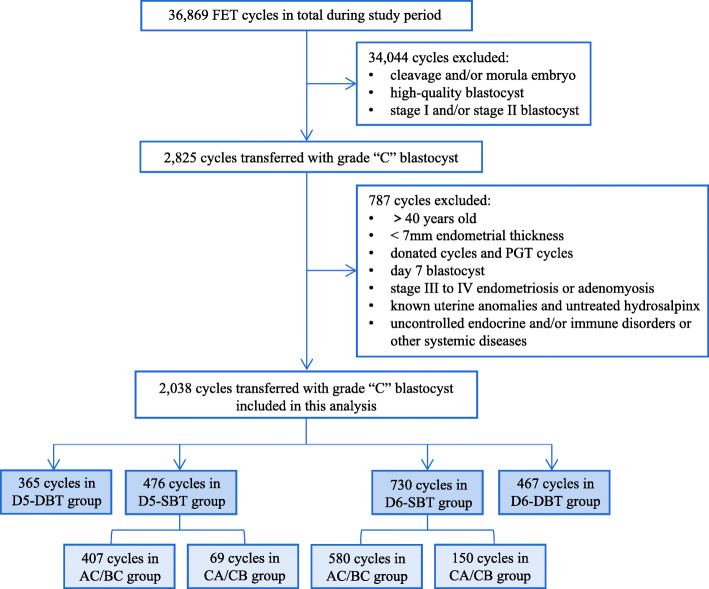
Flowchart of eligibility criteria

During the study period, a total of 36,869 FET cycles were performed at our center. Among these, 2,038 cycles met the study inclusion criteria and were included in the analysis (Fig. 1). All FET cycles were divided into four groups according to the blastocyst development speed and number of transferred embryo. There were 476 cycles included in the D5-SBT group, 365 cycles in the D5-DBT group, 730 cycles in the D6-SBT group, and 467 cycles in the D6-DBT group. Moreover, the patients in the SBT groups were sub-grouped by blastocyst morphology, namely D5-AC/BC, D5-CA/CB, D6-AC/BC, and D5-CA/CB groups.

Comparisons of pregnancy and neonatal outcomes of poor-quality blastocyst FET cycles between SBT and DBT groups stratified by blastocyst development speed are summarized in Table 1. When blastocysts reached the same development speed, there were no statistical differences between the SBT and DBT groups in patients’ age, body mass index (BMI), anti-mullerian hormone (AMH), infertility duration, proportion of endometrial preparation program, endometrial thickness, and rates of implantation, miscarriage, and ectopic pregnancy. For D5 poor-quality blastocysts, the rates of live birth and multiple pregnancy in the DBT group were significantly higher than those in the SBT group (46.03 % vs. 32.35 %, 39.91 % vs. 3.96 %). This conclusion is also valid for the D6 poor-quality blastocyst subgroup; however, the multiple pregnancy rate of the DBT group was approximately 19.21 %, and the live birth rate was only 27.41 %. The vast majority of twins were born prematurely (66.10 % for D5, 61.11 % for D6). In addition, 91.54 % of low birth weight babies came from twins in the D5-DBT group, and 78.26 % were from twins in the D6-DBT group. There was no significant difference between the SBT and DBT groups stratified by twin or singleton in terms of gestational age, newborn height and weight, and proportion of low birth weight infants. When the SBT group was stratified by development speed, there was no difference in patients’ age, BMI, and infertility duration between the D5-SBT and D6-SBT groups. However, rates of clinical pregnancy and live birth in the D5-SBT group were significantly higher than those of the D6-SBT group (42.44 % vs. 20.82 %, 32.35 % vs. 14.25 %).

**Table 1:** Comparisons of poor-quality blastocyst FET cycles between SBT and DBT groups stratified by blastocyst development speed

	D5			D6		
	SBT group	DBT group	*P*	SBT group	DBT group	*P*
Cycles (n)	476	365		730	467	
female age (year)	31.80 ± 4.05	31.40 ± 3.78	0.105	32.92 ± 4.29	32.09 ± 4.20	0.757
BMI (kg/m2)	22.01 ± 3.35	21.58 ± 3.46	0.909	21.83 ± 3.08	22.11 ± 3.24	0.103
AMH (ng/ml)	6.07 ± 4.17	6.75 ± 4.30	0.333	4.82 ± 3.69	4.97 ± 3.96	0.462
Duration of infertility (years)	4.78 ± 3.24	4.71 ± 3.04	0.682	4.86 ± 3.21	4.82 ± 3.20	0.653
Endometrial preparation (%)			0.525			0.447
Natural	38.03 (181/476)	35.89 (131/365)		39.45 (288/730)	37.26 (174/467)	
HRT	61.97 (295/476)	64.11 (234/365)		60.55 (442/730)	62.74 (293/467)	
Endometrial thickness (mm)	8.97 ± 1.39	8.91 ± 1.42	0.387	8.95 ± 1.44	9.05 ± 1.46	0.657
Implantation rate	42.44 (202/476)	41.64 (304/730)	0.785	20.82 (152/730)	22.38 (209/934)	0.445
Pregnancy rate	42.44 (202/476)	59.73 (218/365)	0.000	20.82^#^ (152/730)	37.90 (177/467)	0.000
Multiple pregnancy rate	3.96 (8/202)	39.91 (87/218)	0.000	2.63 (4/152)	19.21 (34/177)	0.000
Early miscarriage rate	22.28 (45/202)	19.72 (43/218)	0.521	27.63 (42/152)	22.03 (39/177)	0.240
Miscarriage rate	22.77 (46/202)	22.48 (49/218)	0.942	30.92 (47/152)	25.99 (46/177)	0.322
Ectopic pregnancy rate	0.99 (2/202)	0.46 (1/218)	0.947	0 (0/152)	1.13 (2/177)	0.501
Live birth rate	32.35 (154/476)	46.03 (168/365)	0.000	14.25^#^ (104/730)	27.41 (128/467)	0.000
Singleton	31.30 (149/476)	29.86 (109/365)	0.654	14.25 (104/730)	23.55 (110/467)	0.000
Twin	1.05 (5/476)	16.16 (59/365)	0.000	0 (0/730)	3.85 (18/467)	0.001
Preterm birth (<37weeks)	9.74 (15/154)	32.14 (54/168)	0.000	6.67 (7/105)	17.19 (22/128)	0.015
Singleton	8.44 (13/154)	8.93 (15/168)	0.877	6.67 (7/105)	8.59 (11/128)	0.584
Twin	1.30 (2/154)	23.21 (39/168)	0.000	0 (0/105)	8.59 (11/128)	0.001
Gestational age (weeks)	38.51 ± 1.75	36.52 ± 3.79	0.000	38.45 ± 1.58	37.79 ± 1.84	0.054
Singleton	38.60 ± 1.74	38.37 ± 1.90	0.428	38.45 ± 1.58	38.29 ± 1.61	0.954
Twin	36.33 ± 0.52	34.83 ± 4.28	0.120	/	36.18 ± 1.60	/
Birth height (mm)	49.75 ± 1.77	47.82 ± 3.65	0.000	49.81 ± 1.82	48.97 ± 2.97	0.001
Singleton	49.89 ± 1.65	49.69 ± 1.70	0.950	49.81 ± 1.82	49.95 ± 1.91	0.813
Twin	46.33 ± 1.21	46.11 ± 4.09	0.197	/	45.55 ± 3.56	/
Birth weight (kg)	3227.16 ± 459.78	2736.0 ± 665.34	0.000	3263.60 ± 538.74	3026.75 ± 599.13	0.101
Singleton	3255.69 ± 442.21	3169.31 ± 468.53	0.944	3263.60 ± 538.74	3207.98 ± 527.65	0.547
Twin	2523.33 ± 318.04	2339.42 ± 564.46	0.294	/	2445.74 ± 420.85	/
Low birth weight (<2500 g)	8.18 (13/159)	31.28 (71/227)	0.000	3.85 (4/104)	15.75 (23/146)	0.003
Singleton	5.37 (8/149)	5.50 (6/109)	0.962	3.85 (4/104)	4.55 (5/110)	0.999
Twin	50.0 (5/10)	55.08 (65/118)	0.999	/	50.0 (18/36)	/

^#^*P* value < 0.05 compared to the D5-SBT group

Comparisons of pregnancy outcomes of single poor-quality blastocyst FET cycles between the AC/BC and CA/CB groups stratified by blastocyst development speed are presented in Table 2. When blastocysts were at the same development speed, there was no statistical difference in patients’ age, BMI, AMH, infertility duration, proportion of endometrial preparation, endometrial thickness, and rates of multiple pregnancy, miscarriage, ectopic pregnancy, and preterm birth between the AC/BC and CA/CB groups. The clinical pregnancy rate (43.73 % vs. 34.78 % for D5; 21.21 % vs. 19.33 % for D6) and live birth rate (33.17 % vs. 27.54 % for D5; 14.66 % vs. 12.67 % for D6) in the AC + BC group was slightly higher than that of the CA + CB group, but the difference was not statistically significant. When blastocysts in the same morphology group were stratified by development speed, there was no significant difference in age, BMI, infertility duration, and endometrial thickness in the D5 group compared with the D6 group. However, the rates of clinical pregnancy and live birth in the D6 group were significantly lower than those in the D5 group.

**Table 2:** Comparisons of FET cycles undergoing SBT between AC/BC and CA/CB groups stratified by blastocyst development speed

	D5				D6		
	AC/BC	CA/CB	*P*		AC/BC	CA/CB	*P*
Cycles (n)	407	69			580	150	
female age (year)	31.87 ± 4.01	31.39 ± 4.23	0.503		32.92 ± 4.30	32.91 ± 4.24	0.877
BMI (kg/m2)	22.01 ± 3.38	22.02 ± 3.17	0.875		21.81 ± 2.97	21.91 ± 3.46	0.242
AMH (ng/ml)	6.13 ± 4.21	5.75 ± 3.94	0.582		4.78 ± 3.63	4.98 ± 3.93	0.887
Duration of infertility (years)	4.75 ± 3.24	4.95 ± 3.26	0.692		4.87 ± 3.21	4.84 ± 3.22	0.721
Endometrial preparation (%)			0.838				0.597
Natural	37.84 (154/407)	62.16 (27/69)			38.97 (226/580)	61.03 (62/150)	
HRT	39.13 (253/407)	60.87 (42/69)			41.33 (354/580)	58.67 (88/150)	
Endometrial thickness (mm)	8.92 ± 1.35	9.27 ± 1.59	0.064		8.98 ± 1.46	8.87 ± 1.37	0.062
Pregnancy rate	43.73 (178/407)	34.78 (24/69)	0.164		21.21^#^ (123/580)	19.33* (29/150)	0.614
Multiple pregnancy rate	3.93 (7/178)	4.17 (1/24)	0.999		2.44 (3/123)	3.45 (1/29)	0.575
Early miscarriage rate	22.47 (40/178)	20.83 (5/24)	0.856		27.64 (34/123)	27.59 (8/29)	0.995
Miscarriage rate	23.03 (41/178)	20.83 (5/24)	0.809		30.08 (37/123)	34.48 (10/29)	0.645
Ectopic pregnancy rate	1.12 (2/178)	0 (0/24)	0.999		0 (0/123)	0 (0/29)	/
Live birth rate	33.17 (135/407)	27.54 (19/69)	0.355		14.66^#^ (85/580)	12.67* (19/150)	0.535
Singleton	32.19 (131/407)	26.09 (18/69)	0.312		14.66 (85/580)	12.67 (19/150)	0.535
Twin	0.98 (4/407)	1.45 (1/69)	0.545		0 (0/580)	0 (0/150)	/
Preterm birth (<37weeks)	8.89 (12/135)	15.79 (3/19)	0.592		8.14 (7/86)	0 (0/19)	0.436
Singleton	8.15 (11/135)	10.53 (2/19)	0.999		8.14 (7/86)	0 (0/19)	0.436
Twin	0.74 (1/135)	5.26 (1/19)	0.232		0 (0/86)	0 (0/19)	/

^#^*P* value < 0.05 compared to the D5-AC/BC group

^*^P value < 0.05 compared to the D5-CA/CB group

Table 3 displays the effect of blastocyst with ICM “C” or TE “C” on neonatal outcomes of single blastocyst transfer FET cycles. Considering the small number of twins, only singleton data were included in the analysis. When blastocysts in the same morphology group were stratified by development speed, they were divided into four groups. There was no significant difference in the gestational age, newborn weight, and height, and the proportion of low birth weight infants among those groups.

**Table 3:** Effect of SBT with poor-quality blastocyst on neonatal outcomes

	D5		D6		
	AC/BC	CA/CB	AC/BC	CA/CB	*P*
Cycles (n)	131	18	85	19	
Gestational age (weeks)	38.58 ± 1.75	38.78 ± 1.70	38.52 ± 1.67	38.35 ± 0.93	0.893
Birth height (mm)	49.84 ± 1.65	50.22 ± 1.66	49.83 ± 1.69	49.82 ± 2.40	0.839
Birth weight (kg)	3241.58 ± 435.86	3357.61 ± 486.69	3271.43 ± 561.47	3265.29 ± 422.09	0.810
Low birth weight(<2500 g)	5.34 (7/131)	5.56 (1/18)	3.53 (3/85)	5.26 (1/19)	0.849

Note: only singleton data were included in the analysis

## Discussion

This study explored the effect of poor-quality blastocyst development speed and morphology on pregnancy and neonatal outcomes, and ultimately provided references for poor-quality blastocyst transfer strategy in clinical practice. This study suggested that single D5 blastocyst could be preferentially recommended to patients with only poor-quality because of an acceptable live birth rate and significantly reduced multiple pregnancy rate compared with DBT. DBT was considered for D6 blastocysts to improve the live birth rate. When blastocyst reach the same development speed, the transfer of blastocyst with ICM “C” or blastocyst with TE “C” had no impact on pregnancy and neonatal outcomes.

Multiple pregnancy is a common iatrogenic complication of IVF-ET. Single embryo transfer is an effective and powerful measure to avoid iatrogenic multiple pregnancy. The strategy of single high-quality blastocyst transfer has been gradually accepted by reproductive clinicians because of its acceptable live birth rate and the significantly reduced multiple pregnancy rate, which could significantly improve perinatal outcomes [6]. Meanwhile, Wang et al. showed that the live birth rate is approximately 47.6 % and the multiple pregnancy rate is 30.7 % for DBT composed of high-quality and poor-quality blastocysts [5]. Our previous study suggested that the live birth rate of single good-quality D5 blastocyst was about 54.25 % and the multiple pregnancy rate was 3.52 % for patients under 35 years old. And the live birth rate of DBT composed of good-quality and poor-quality blastocysts was 64.08 %, and the multiple rate was 49.66 % [9]. These studies indicated that the transfer of an additional poor-quality blastocyst does not negatively affect the implantation potential of high-quality blastocysts. Nevertheless, the addition of poor-quality blastocysts contributes to both live birth and multiple births. For patients without high-quality blastocysts, the strategy of DBT is generally recommended to improve the live birth rate. Research has reported that the multiple pregnancy rate of DBT in poor-quality blastocysts is still as high as 33 % [8]. Moreover, our previous study revealed the multiple rate of double poor-quality blastocysts was as high as 50 % [9]. Dobson et al. showed that the live birth rate of single poor-quality blastocysts was similar to that of double poor-quality blastocysts, but the multiple pregnancy rate was significantly decreased in patients undergoing the FET cycle [8]. Our results of this study are different from this conclusion and showed that the rates of live birth and multiple pregnancy of DBT are significantly higher than those of SBT for poor-quality blastocysts. The live birth rate of the D5-SBT group could reach 32.35 %, and 14.25 % in the D6-SBT group. Therefore, the strategy of single poor-quality D5 blastocyst transfer can be suggested to reduce the multiple pregnancy rate. For poor-quality D6 blastocysts, there are few reports on whether single embryo transfer is also recommended.

The comparison of pregnancy outcomes between D5 and D6 blastocysts remains controversial. The differences in study results may be caused by blastocyst culture systems, frozen–thawed methods, different blastocyst culture strategies and methods, and highly subjective blastocyst scoring methods in different centers [12]. Ferreux et al. [13] revealed that the pregnancy outcomes following frozen-thawed blastocyst transfer are significantly lower with D6 than with D5 blastocyst regardless of embryo quality. Shen et al. [10] found that blastocyst frozen days (day 5 or 6) had no impact on live birth rate for AA/AB/BA blastocysts; however, those frozen on day 5 had significantly better live birth rates than those frozen on day 6 for BB/BC/CB blastocysts. The results of this study are consistent with this and show that the rates of clinical pregnancy and live birth of patients in the D5-SBT group were significantly higher than those of the D6-SBT group for poor-quality blastocysts. Kroener et al. [14] found that delayed blastulation is not associated with increased aneuploidy rates, but this conclusion has not been further compared and analyzed when stratified by blastocyst quality. Yang et al. [12] performed preimplantation genetic screening (PGS) on 237 blastocysts and found that blastocyst development speed had no effect on the euploid rate for high-quality blastocysts (55.2 % vs. 55.3 %, *P* > 0.05). The rates of euploid and clinical pregnancy in the D5 group were higher than those in the D6 group for poor-quality blastocysts (P > 0.05). Although there was no statistical difference, it may be the reason for the lower pregnancy rate of the D6 poor-quality blastocysts. Similarly, another study revealed that Day 5 euploid good-quality blastocysts had no significant difference in implantation potential compared with similarly graded Day 6 euploid blastocysts (80.43 % vs. 75.0 %). And patients transferred with Day 5 euploid poor-quality blastocysts had a nonsignificant trend toward a higher implantation rate than similarly graded Day 6 blastocysts (55.77 % vs. 42.47 %) [15]. These findings suggest that blastocyst development speed may have little predictive value for the developmental potential of high-quality blastocysts but may have a certain predictive value for poor-quality blastocysts. The result of this study showed that the pregnancy rate of single poor-quality D6 blastocyst without PGT technique is 20.82 %. Therefore, for patients without performing PGT, DBT is recommended to increase the live birth rate for poor-quality D6 blastocysts, and the patient needs to be informed that there is a 14.06 % (18/128) risk of multiple births.

The blastocyst grading scheme is usually based on the Gardner grading system. The parameters included the expansion degree and individual evaluation of the ICM and TE. The effect of blastocyst morphology parameters on pregnancy outcomes is controversial. Du et al. [16] found that the blastocyst expansion degree was the only indicator that predicted the live birth rate in both fresh and vitrified-warmed single blastocyst transfer cycles, and neither ICM nor TE grade was correlated with live birth rate. Subira et al. [17] showed that ICM, rather than TE, has a better predictive value for live birth in fresh SBT. Chen et al. [18] suggested that TE grading, but not ICM grading, was significantly associated with clinical pregnancy rate and live birth rate in FET cycles in a Chinese population. The inconsistency of these research results brought confusion to the blastocyst transfer strategy in clinical practice, whether we choose the blastocyst with a better ICM or the blastocyst with a better TE when selecting a single poor-quality blastocyst for transferring. This study found that there was no significant difference in the rates of clinical pregnancy, live birth, and early miscarriage between the AC/BC and CA/CB groups when blastocysts were at the same development speed. The early miscarriage rate of each group reached more than 20 %, suggesting that both ICM and TE play an extremely important role in the whole process of embryo development.

In this study, patients with singleton births undergoing SBT were divided into four groups based on blastocyst development speed and morphology. The results showed that there was no significant difference in the gestational age, neonatal height and weight, and the proportion of low birth weight among the four groups. Although the live birth rate of D6 blastocysts is significantly lower than that of the D5 group whether for AC/BC or CA/CB blastocysts, but once persistent pregnancy is achieved, similar neonatal outcomes can be obtained. However, it cannot be ruled out that there is no statistically significant difference due to the small sample size. Additionally, Oron et al. [19] showed that pregnancy resulting from poor-quality embryos, whether at the cleavage or blastocyst stage, did not result in any obvious increased risk of adverse obstetric or perinatal outcomes compared with good-quality embryos. Similarly, Bouillon et al. [20] suggested that the main obstetric and perinatal outcomes of singletons after transfer of blastocysts with poor morphological characteristics were not associated with increased adverse obstetric and perinatal events. Therefore, the implementation of single poor-quality blastocyst transfer can reduce multiple pregnancy, improve perinatal maternal and infant outcomes, and obtain a similar perinatal outcome to that of high-quality embryos. This information could be important to reassure couples who conceive following the transfer of poor-quality embryos.

There are some limitations to this study. First, the sample size included in each group is uneven; therefore, the results of the study may be biased, and further research is needed to confirm the conclusion of this study. In addition, considering that ICM develops into the fetus, a lower ICM score may affect fetal development in the future. Therefore, embryo transfer strategies tend to choose blastocysts with better ICM for transfer in clinical practice, which may also be an important reason for the uneven sample size. However, as far as we know, the number of patients included in this study among each group was larger than that of other similar studies, making the results from this study valuable for guiding the transfer strategy of poor-quality blastocysts.

## Conclusions

When blastocysts reach the same grade, we should focus on embryo development speed before the transfer for poor-quality blastocysts because the pregnancy outcomes of D5 blastocysts are significantly better than those of D6 blastocysts. For poor-quality D5 blastocysts, SBT could be recommended because of the acceptable live birth rate and significantly reduced multiple pregnancy rates compared to DBT. For poor-quality D6 blastocysts, DBT is recommended to improve pregnancy outcomes. Meanwhile, once a continuous pregnancy is reached, the blastocyst development speed and morphology do not affect neonatal outcomes.

## Data Availability

The datasets used and/or analyzed during the current study are available from the corresponding author upon reasonable request.

## Abbreviations

AMH: Anti-mullerian hormone
ANOVA: One-way analysis of variance
ART: Assisted reproductive technique
BMI: Body mass index
DBT: Double blastocyst transfer
FET: Frozen embryo transfer
HRT: Hormone replacement therapy
ICM: Inner cell mass
ICSI: Intracytoplasmic sperm injection
IVF-ET: In vitro fertilization and embryo transfer
LBR: Live birth rate
NC: Natural cycle
PGT: Preimplantation genetic testing
PGS: Preimplantation genetic screening
SBT: Single blastocyst transfer
SD: Standard deviation
SPSS: Statistical package for social science
TE: Trophectoderm
